# Polystyrene Nanoplastics Increase Macrophage Bactericidal Activity Through a Mechanism Involving Reactive Oxygen Species and Itaconate

**DOI:** 10.3390/nano16020105

**Published:** 2026-01-13

**Authors:** Seyedeh Safoora Moosavi, Hamlet Acevedo Ospina, Albert Descoteaux

**Affiliations:** INRS—Centre Armand-Frappier Santé Biotechnologie, Laval, QC H7V1B7, Canada; safoora.moosavi@inrs.ca (S.S.M.); hamlet.acevedo@inrs.ca (H.A.O.)

**Keywords:** polystyrene nanoplastics, macrophages, reactive oxygen species, itaconate, bactericidal activity

## Abstract

Nanoplastics are persistent environmental pollutants with potential risks to human health. Due to their small size, nanoplastics are internalized by macrophages, potentially altering their function. In this study we found that, in macrophages, 50 nm polystyrene nanoplastics were predominantly present in endosomes, lysosomes, and in the endoplasmic reticulum. Internalization of polystyrene nanoplastics increased the bactericidal activity of macrophages, which was inhibited by the NADPH oxidase inhibitor diphenyleneiodonium. Consistently, measurements of cellular and mitochondrial reactive oxygen species by flow cytometry revealed that polystyrene nanoplastics induced reactive oxygen species production in macrophages. In contrast, internalization of polystyrene nanoplastics reduced the levels of nitric oxide released by macrophages in response to *E. coli*. Internalization of polystyrene nanoplastics followed by the addition of *E. coli* induced high expression levels of the aconitate decarboxylase 1 gene. In the absence of this gene, killing of *E. coli* by macrophages exposed to polystyrene nanoplastics was significantly attenuated with respect to control macrophages, indicating a role for the mitochondrial metabolite itaconate in the increased bactericidal activity of macrophages exposed to polystyrene nanoplastics. Collectively, our results indicate that exposure of macrophages to polystyrene nanoplastics increases their bactericidal activity through the production of reactive oxygen species and of itaconate.

## 1. Introduction

The environmental and health impacts of plastic-derived micro- and nanoparticles is a major concern [1,2]. Plastic particles smaller than 1 µm, known as nanoplastics (NPs), constitute a concerning class of pollutants due to their small size, environmental persistence, and potential for bioaccumulation [3]. Their ubiquity and persistence raise significant concerns regarding their impact on human health, especially through inhalation, ingestion, or dermal exposure [4,5]. Once inside the body, nanoplastics may cross physiological barriers, accumulate in tissues, and interact with various cell types, potentially inducing toxic or immunomodulatory effects [6]. Among those are polystyrene nanoplastics (PS-NPs), which are commonly found in consumer products and are prevalent in air, water, and soil [7].

Professional phagocytes such as macrophages play a major role in innate immunity owing to their ability to engulf and eliminate pathogens, clear cellular debris, and orchestrate inflammatory responses [8,9,10]. Given their role in surveillance and immune regulation, macrophages are among the first cell types to encounter foreign particles, including NPs [11]. Internalization of NPs by macrophages can interfere with normal cellular functions through physical interactions, inducing oxidative stress, altering signaling pathways, modulating gene expression, metabolism, and inflammation [12,13,14]. Therefore, better understanding how macrophages respond to PS-NPs is essential for assessing the potential health impact of being exposed to these particles [15,16].

Despite increasing recognition of NPs as environmental and health hazards, their specific interactions with immune cells, particularly their intracellular localization and their effects on anti-microbial and inflammatory responses, remain poorly understood. Prior studies have suggested that NPs can localize within cellular compartments, such as lysosomes, endosomes, and the endoplasmic reticulum (ER), potentially disrupting key cellular functions [17,18]. However, the extent and implications of such intracellular trafficking in macrophages are still being elucidated. Moreover, while oxidative stress contributes to NP-induced toxicity [19], its direct relationship with immune cell function, including microbicidal activity, remains poorly understood.

PS is highly prevalent in the environment and PS-NPs have been found in various tissues in mammals and humans [20,21]. For these reasons, the effects of PS-NPs on cells, tissues, and systems have received considerable attention [14,18,21,22,23]. In the present study, we aimed at examining the functional impact of PS-NPs on macrophages. Our findings indicate that PS-NPs localize in various intracellular compartments and that they increase macrophage bactericidal activity through the generation of reactive oxygen species (ROS) and the synthesis of the mitochondrial metabolite itaconate. These results deepen our understanding of NPs–immune cell interactions by providing novel insights on the impact of NPs on immune and metabolic processes in macrophages.

## 2. Materials and Methods

### 2.1. Ethics Statement

Work with animals was performed according to protocols 2112-01 and 2110-04, which were approved by the Comité Institutionnel de Protection des Animaux of the Institut national de la recherche scientifique—Centre Armand-Frappier Santé Biotechnologie. These protocols respect procedures on animal practice promulgated by the Canadian Council on Animal Care, described in the Guide to the Care and Use of Experimental Animals.

### 2.2. Characterization of PS-NPs

Commercial 50 nm fluorescent polystyrene nanoparticles (PS-NPs) were obtained from Thermo Fisher Scientific (Waltham, MA USA) (Fluoro-Max Dyed Blue Aqueous Fluorescent Particles, lot 263393). These NPs were supplied as aqueous suspensions at a concentration of 1% solids by weight in deionized water, containing trace amounts of surfactant and preservative to prevent aggregation and maintain colloidal stability. The PS-NPs had a density of 1.06 g/cm^3^ and a refractive index of 1.59. The particles were internally dyed with fluorescent dye. PS-NPs at a concentration of 1 μg/mL in either HPLC water or DMEM supplemented with 10% heat-inactivated FBS were characterized by Dynamic Light Scattering (DLS) using the Zetasizer Nano-ZS Zen 3600 (Malvern Instruments, Westborough, MA, USA). The size, hydrodynamic diameter, polydispersity index (PDI), and ζ potential were calculated with the instrument software.

### 2.3. Antibodies

The primary antibodies used in this study were rabbit anti-P115, rabbit anti-calnexin, mouse anti-Rab7 (Invitrogen–Molecular Probes, Carlsbad, CA, USA), and rat anti-LAMP-1 monoclonal antibody (clone 1D4B), developed by J. T. August and obtained from the Developmental Studies Hybridoma Bank (University of Iowa, Iowa City, IA, USA), supported by the National Institute of Child Health and Human Development. Secondary antibodies for immunofluorescence were as follows: Alexa Fluor 488–conjugated anti-mouse IgG, Alexa Fluor 647–conjugated anti-rabbit IgG, and Alexa Fluor 647–conjugated anti-rat IgG (Invitrogen–Molecular Probes, Carlsbad, CA, USA).

### 2.4. Macrophage Culture

The murine LM-1 immortalized bone-marrow-derived macrophage cell line (iBMMs) [24] was maintained in Dulbecco’s Modified Eagle Medium (DMEM; high glucose, Gibco Life Technologies, Grand Island, NY, USA) supplemented with 10% heat-inactivated fetal bovine serum (FBS; Gibco), 1% penicillin–streptomycin (Gibco), and 2 mM L-glutamine (complete DMEM) and was incubated at 37 °C in a humidified atmosphere with 5% CO_2_. These iBMMs were generated by infecting bone marrow cells obtained from C3H mice with the J2 retrovirus [24]. Murine BMMs were generated as previously described [25]. Femurs and tibias of wild-type (WT) and *Acod1* knockout (*Acod1*^−/−^) C57BL/6 male and female mice aged 8 to 12 weeks (JAX) were collected under sterile conditions, and bone marrow was flushed using HBSS through a 26½ G needle attached to a 10 mL syringe. The extracted marrow was passed through a 70 µm cell strainer and centrifuged at 2000 RPM for 5–6 min at 4 °C. Red blood cells were lysed using ammonium chloride lysis buffer, followed by a wash in HBSS and a second centrifugation. The resulting cell pellet was resuspended in complete DMEM supplemented with 10% heat-inactivated FBS, 10 mM HEPES (pH 7.4), 100 IU/mL penicillin, 100 μg/mL streptomycin, and 15% (*v*/*v*) L929 cell-conditioned medium (as a source of macrophage colony-stimulating factor, M-CSF). Cells were plated in non-adherent dishes and incubated at 37 °C in a humidified atmosphere containing 5% CO_2_. Medium was supplemented with fresh L929-conditioned medium on days 3, 5, and 7 to promote macrophage differentiation. On day 8, BMMs were harvested by gentle scraping in cold HBSS containing HEPES and antibiotics, followed by centrifugation. To render the BMMs quiescent, cells were cultured for an additional 24 h in complete DMEM lacking L929-conditioned medium prior to downstream experiments.

### 2.5. Treatment of Cells with PS-NPs

Cells were seeded in either 96-well plates (200 μL/well), 24-well plates (500 μL/well), or 6-well plates (2 mL/well) in complete DMEM and were incubated for 18 h at 37 °C in a humidified incubator with 5% CO_2_. Cells were then treated with various stimuli depending on the experimental conditions, including PS-NPs. Unless otherwise stated, iBMMs and BMMs were incubated with 1 μg/mL PS-NPs for 18 h prior to additional treatments. All treatments were carried out in fresh medium for the indicated time points prior to downstream analyses.

### 2.6. MTT Assay for Cell Viability

Viability of iBMMs exposed to PS-NPs was ascertained using the MTT assay [26]. Cells (1.5 × 10^4^) were seeded into 96-well plates and incubated overnight to allow the cells to adhere. The cells were then treated for 24 h with various concentrations of PS-NPs (0, 1, 2, 7, 5, and 10 μg/mL) to assess cytotoxicity. After the exposure period, cells were incubated with a 0.5 mg/mL MTT solution (Thiazole Blue Tetrazolium Bromide) at 37 °C for 2 h so that viable cells could convert MTT to purple formazan crystals. Following incubation, the MTT-containing medium was replaced with 100 µL of dimethyl sulfoxide for the dissolution of formazan crystals and plates were read at 570 nm on a microplate reader. Cell viability was calculated by comparing absorbance values of PS-NPs-treated cells with respect to the control untreated group.

### 2.7. Confocal Microscopy

iBMMs were seeded in 24-well plates containing sterile microscope coverslips and treated with PS-NPs as described above. After treatment, cells were washed with phosphate-buffered saline (PBS), fixed with 3.7% paraformaldehyde for 30 min at room temperature, and permeabilized with 0.1% Triton X-100 (Sigma Aldrich, Oakville, ON, Canada) for 5 min. Following permeabilization, samples were blocked in 10% BSA (Sigma Aldrich) in PBS for 1 h. Cells were then incubated for 1 h at room temperature with the following primary antibodies diluted in 1% BSA/PBS: rat LAMP1 (1:500), mouse Rab7 (1:600), rabbit P115 (1:500), and rabbit Calnexin (1:500). After washing, cells were incubated for 1 h with the appropriate secondary antibodies at 1:500 dilution: anti-rat Alexa Fluor 647, anti-mouse Alexa Fluor 488, and anti-rabbit Alexa Fluor 647. After three washes with PBS, coverslips were mounted individually onto glass slides using Fluoromount-G (Invitrogen). Images were acquired with a Zeiss LSM780 confocal microscope (Jena, Germany) equipped with a Plan-Apochromat X 63 oil-immersion objective (NA 1.4) in differential interference contrast (DIC) mode. Images were acquired in sequential scanning mode and were processed using ZEN 2012 software (Carl Zeiss Microimaging, Rostock, Germany). For each condition, a minimum of 25 cells were analyzed using ZEN 2012 or Icy image analysis software (v2.5.4.0).

### 2.8. Bactericidal Assay

iBMM and BMM derived from WT and *Acod1*^−/−^ mice treated with 1 μg/mL PS-NPs were incubated with non-opsonized *Escherichia coli* DH1 (OD600 = 0.6) at a bacteria-to-macrophage ratio of 20:1. Bacteria in 20 μL PBS were added to wells containing macrophages; plates were centrifuged at 1000× *g* for 1 min and incubated at 37 °C for 20 min. Wells were washed four times with PBS and remaining non-ingested *E. coli* were eliminated by adding 5 mg/mL gentamicin (Life Technologies, Grand Island, NY, USA) to complete DMEM medium for 20 min (zero time point) or for an additional 4 h [27]. Bacterial killing was determined by enumerating colonies on agar plates. Where indicated, iBMM and BMM were incubated for 1 h with 10 μM of the NADPH oxidase inhibitor diphenyleneiodonium (DPI) prior to the addition of *E. coli*. Results were expressed as the log_10_ of colony-forming units per mL (CFU/mL).

### 2.9. ROS Measurement

iBMMs unexposed to PS-NPs were left untreated (control) or were incubated with either zymosan (5:1) or *E. coli* (20:1) for 1 h in the presence of either 5 μM CellROX Deep Green Reagent (Thermo Fisher Scientific, Waltham, MA USA) or 5 μM MitoSOX Mitochondrial Superoxide Indicators Red (Thermo Fisher Scientific). iBMMs exposed for 18 h to 1 μg/mL PS-NPs were incubated or not with *E. coli* (20:1) for 1 h in the presence of either 5 μM CellROX or 5 μM MitoSOX. Additionally, iBMMs were exposed for 1 h to 1 μg/mL PS-NPs in the presence of either 5 μM CellROX or 5 μM MitoSOX. Subsequently, cells were washed twice with PBS and were collected by scraping in HBSS, fixed for 10 min in 4% PFA prior to two washes with PBS. Samples were acquired in the APC channel on an LSRFortessa SORP cytometer with the BD FACSDiva6.2 software (BD Biosciences, Mississauga, ON, Canada) [28]. Results are presented as mean fluorescence intensity.

### 2.10. Nitric Oxide Production

Nitric oxide (NO) production was determined by measuring nitrite using the Griess reagent assay [29]. Briefly, 100 μL of cell culture supernatants were mixed with an equal volume of Griess reagent (1% sulfanilamide and 0.1% N-(1-naphthyl) ethylenediamine dihydrochloride) in a 96-well plate and incubated at room temperature for 10 min. A microplate reader was used to read absorbance at 570 nm. Sodium nitrite was used to generate a standard curve to calculate nitrite concentration. Results are expressed in μM.

### 2.11. Gene Expression Analysis by RT-qPCR

Total RNA was extracted from iBMMs subjected to various experimental conditions, including untreated control and treatment with 100 ng/mL lipopolysaccharide (LPS), 1 μg/mL PS-NPs, *E. coli* (5:1), and co-treatment with PS-NPs and *E. coli*, at 8 h and 18 h post-treatments. RNA extraction was performed using the RNeasy^®^ Mini Kit (Qiagen, Toronto, ON, Canada). A NanoDrop^TM^ spectrophotometer (Thermo Fisher Scientific) was used to determine RNA concentration and purity, with acceptable A260/A280 ratios ranging between 1.8 and 2.0. Agarose gel electrophoresis was used to visualize RNA integrity in selected samples. First-strand complementary DNA (cDNA) was synthesized from 500 ng of total RNA using the iScript^TM^ cDNA Synthesis Kit (Bio-Rad Laboratories, Hercules, CA, USA). qPCR was conducted using the iTaq^TM^ Universal SYBR^®^ Green Supermix (Bio-Rad) on either the Stratagene Mx3000P or QuantStudio^TM^ 3 Real-Time PCR System (Applied Biosystems, Waltham, MA, USA). Gene expression levels of *Acod1* were quantified using gene-specific primers, with *Rps29* used as the housekeeping gene for normalization. Relative expression was calculated using the 2^−ΔΔCt^ method, comparing treated samples to control conditions after normalization to the housekeeping gene. The following primers were used: *Acod1*-F: 5′-GCAACATGATGCTCAAGTCTG-3′; *Acod1*-R: 5′-TGCTCCTCCGAATGATACCA-3′; *Rps29*-F: 5′-CACCCAGCAGACAGACAAACTG-3′; and *Rps29*-R: 5′-GCACTCATCGTAGCGTTCCA-3′.

### 2.12. Statistical Analysis

All data resulted from the average of at least three independent experiments performed in triplicates. The GraphPad Prism 7 software was used to analyze data; statistical analysis was performed with the two-way ANOVA with Tukey’s multiple comparison test unless stated otherwise. Statistical significance was defined as a * *p* ≤ 0.05, ** *p* ≤ 0.01, and *** *p* ≤ 0.001.

## 3. Results

### 3.1. Intracellular Localization of PS-NPs in iBMM

The 50 nm fluorescent PS-NPs used in this study were characterized by DLS. In water, they had an average size of 50.83 nm, a hydrodynamic diameter of 47.95 nm, a polydispersity index (PDI) value of 0.106, and a ζ-potential of −14.3 mV (Table 1). In DMEM supplemented with 10% FBS, the values were altered by the presence of components which may include extracellular vesicles [30].

DLS analyses of the 50 nm fluorescent PS-NPs in DMEM supplemented with 10% FBS revealed the presence of two major peaks for particle size. Particles in Peak 1 had an average size of 13.92 nm and those in Peak 2 had an average size of 57.87 nm. The latter may represent the 50 nm fluorescent PS-NPs. DLS analyses of the DMEM supplemented with 10% FBS showed only one peak at 22.27 nm (Table 2).

**Figure 1:**
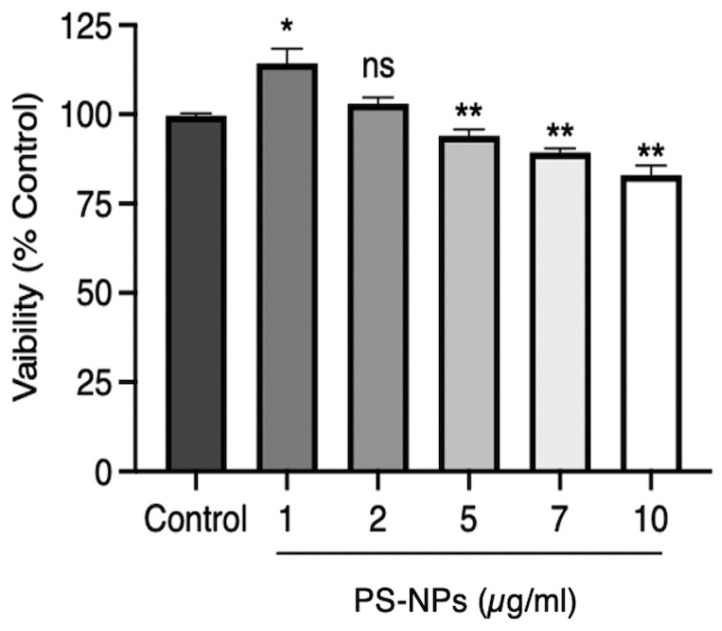
Cell viability was assessed in iBMMs exposed for 24 h to 50 nm PS-NPs at concentrations ranging from 0 to 10 µg/mL PS-NPs. Cell viability is expressed relative to the control iBMMs. Results are representative of three independent experiments performed in triplicate. Data are presented as mean ± SD of triplicate experiments. * *p* < 0.05, ** *p* < 0.01. ns, not significant.

Prior to studying their intracellular localization and their impact on macrophage function, we first exposed iBMMs to PS-NPs at various concentrations for 24 h to assess their toxicity. As illustrated in Figure 1, PS-NPs were slightly cytotoxic at concentrations of 5 µg/mL and above.

**Figure 2:**
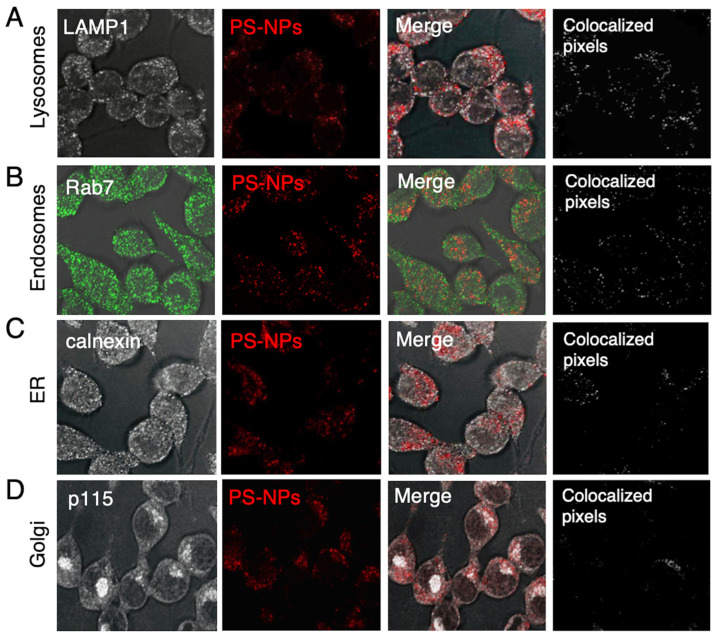
Intracellular localization of PS-NPs in iBMMs. iBMMs were incubated with 1 μg/mL fluorescent PS-NPs for 24 h. Colocalization of PS-NPs (red) with (**A**) LAMP1 (white), (**B**) Rab7 (green), (**C**) calnexin (white), and (**D**) p115 (white) was evaluated by confocal immunofluorescence microscopy. Colocalized pixels are shown in white. Representative images from 3 independent experiments are shown.

We therefore decided to use 1 µg/mL of PS-NPs for our experiments, a concentration that did not affect iBMM viability. To assess the sub-cellular localization of PS-NPs 24 h post-internalization, we used confocal immunofluorescence microscopy. As shown in Figure 2, fluorescent PS-NPs colocalized with the lysosomal protein LAMP1 (Figure 2A), the endosomal protein Rab7 (Figure 2B), as well as the ER protein calnexin (Figure 2C). On the other hand, we did not observe significant colocalization of PS-NPs with the Golgi protein p115 (Figure 2D). These results indicate that PS-NPs are internalized by macrophages, with accumulation predominantly in lysosomes and endosomes and, to a lesser extent, in the ER. These findings underscore the endo-lysosomal pathway as a route for intracellular PS-NPs trafficking.

### 3.2. PS-NPs Increase the Bactericidal Activity of Macrophages

**Figure 3:**
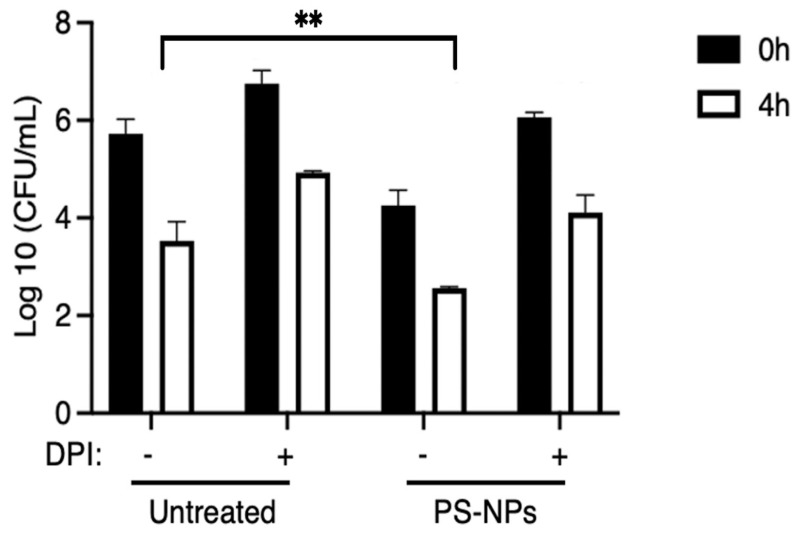
The bactericidal activity of iBMMs (untreated or exposed to 1 μg/mL PS-NPs for 18 h) was determined as described in Materials and Methods. Where indicated, 10 μM DPI was added 1 h prior to the addition of *E. coli*. Bactericidal activity was determined by counting CFU at 0 h and 4 h post-treatment with gentamycin. Data are presented as mean ± SEM and are representative of three independent experiments performed in triplicate. ** *p* < 0.01.

Given the importance of the endo-lysosomal compartments in the killing of internalized bacteria [31,32], we tested the hypothesis that accumulation of PS-NPs in those compartments may alter the bactericidal capacity of macrophages. To this end, iBMM incubated in the presence or absence of 1 µg/mL PS-NPs for 18 h were fed with *E. coli*. Since ROS play a major role in bacterial killing [33], we determined their contribution using diphenyleneiodonium (DPI), a potent inhibitor of the NADPH oxidase [34]. Results shown in Figure 3 indicate that exposure of iBMM to PS-NP significantly increased the killing of *E. coli*. Furthermore, pretreatment of iBMM with DPI markedly attenuated bacterial clearance, suggesting that the enhanced bactericidal activity induced by PS-NPs is, at least in part, dependent on NADPH oxidase-mediated ROS production.

**Figure 4:**
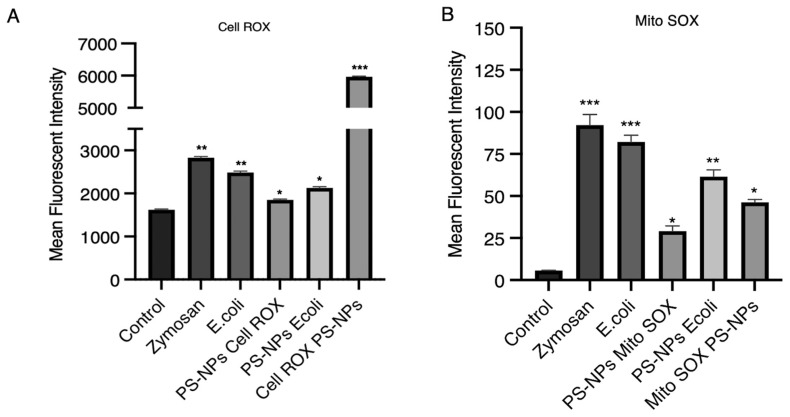
ROS levels in iBMMs exposed to PS-NPs. (**A**) Total and (**B**) mitochondrial ROS produced by iBMMs were assessed by flow cytometry using CellROX Deep Green Reagent and MitoSOX^TM^ Mitochondrial probe, respectively. iBMMs unexposed to PS-NPs were left untreated (control) or were incubated with either zymosan or *E. coli* for 1 h in the presence of either CellROX or MitoSOX. iBMMs exposed for 18 h to 1 μg/mL PS-NPs were incubated or not with *E. coli* for 1 h in the presence of either CellROX or MitoSOX (PS-NPs CellROX or PS-NPs MitoSOX and PS-NPs *E.coli*). Additionally, iBMMs were exposed for 1 h to 1 μg/mL PS-NPs in the presence of either CellROX or MitoSOX (CellROX PS-NPs or MitoSOX PS-NPs). Mean fluorescence intensity (MFI) for each condition relative to the untreated control is shown. Data are presented as mean MFI ± SEM and are representative of three independent experiments performed in triplicate. * *p* < 0.05, ** *p* < 0.01, *** *p* < 0.001.

The fact that bacterial clearance was not fully inhibited by DPI suggests that other components of the macrophage microbicidal machinery might contribute to *E. coli* killing, regardless of the presence of PS-NPs. To determine the extent of ROS produced by untreated and PS-NPs-treated iBMM, we next quantified ROS produced by control and PS-NPs-treated iBMM exposed to *E. coli*. To this end, we measured by flow cytometry total cell ROS using CellROX and mitochondrial ROS using MitoROS. As expected, iBMM exposed to zymosan or *E. coli* produced high levels of total cellular ROS, whereas iBMM exposed to PS-NPs for 18 h produced slightly higher levels of cellular ROS than control iBMM (Figure 4A). Of note, initial exposure of iBMM to PS-NPs induced high levels of total cellular ROS, which declined over 24 h. In contrast, iBMM incubated for 18 h with PS-NPs produced less cellular ROS than untreated iBMM upon exposure to *E. coli*. In the case of mitochondrial ROS, we observed that PS-NPs alone induced lower levels with respect to zymosan and *E. coli* and that iBMM exposed for 18 h to PS-NPs produced less mitochondrial ROS than untreated iBMM in response to *E. coli* (Figure 4B). Hence, although ROS contribute to the enhanced bacterial killing observed in iBMM pretreated with PS-NPs, our results suggest that ROS-independent mechanism(s) may also contribute to the bactericidal activity of PS-NPs-treated iBMM.

### 3.3. PS-NPs Impair Nitric Oxide Production

**Figure 5:**
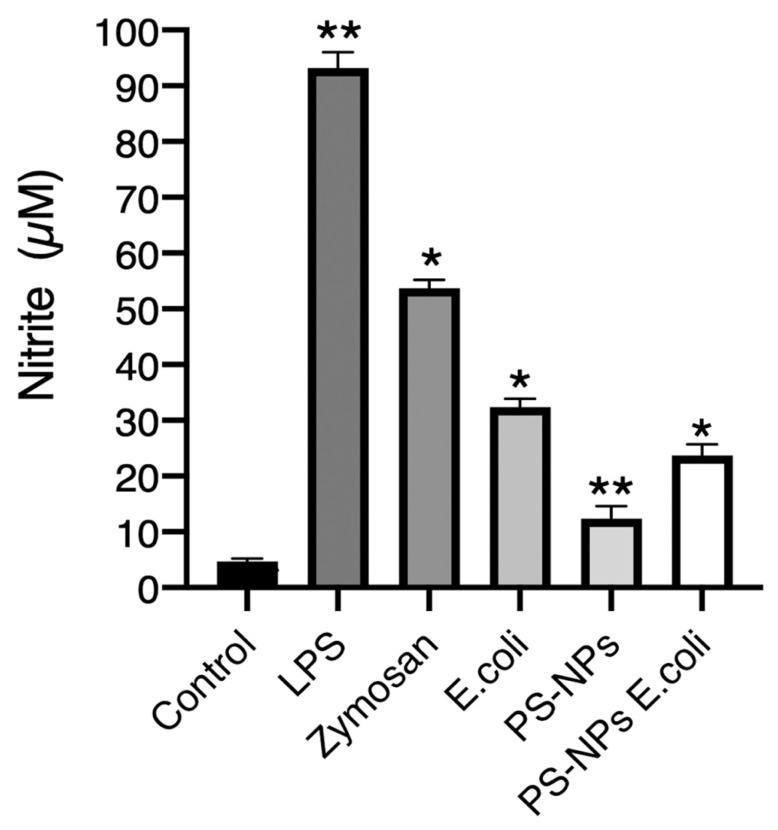
Exposure to PS-NPs impairs NO production. iBMM untreated or exposed to PS-NPs for 18 h were incubated with either LPS (100 ng/mL), zymosan (5:1), or *E*. *coli* (20:1) for 18 h prior to the determination of nitrite levels. Results are representative of three independent experiments performed in triplicate. Data are represented as mean ± SEM. * *p* < 0.05, ** *p* < 0.01.

Nitric oxide (NO) is a potent microbicidal molecule [35]. To examine its potential role in the enhanced bacterial killing observed in iBMM pretreated with PS-NPs, we determined its production in iBMM treated with either 100 ng/mL LPS, zymosan (5:1), or *E. coli* (20:1) for 18 h and in iBMM exposed to PS-NPs and incubated or not with *E. coli* for 18 h by measuring nitrites in cell supernatants. As expected, very low levels of nitrites were present in the supernatants of untreated cells, indicating minimal inducible nitric oxide synthase activity in the absence of stimulation, whereas exposure of iBMM to LPS, zymosan, and *E. coli* for 18 h led to high levels of nitrites in supernatants (Figure 5). These results indicate that these stimuli induced the production of NO in iBMM. In contrast, exposure to PS-NPs stimulated the production of low NO levels, as determined by the levels of nitrites in supernatants. Pretreatment of iBMM with PS-NPs resulted in a reduced production of NO in response to *E. coli*, suggesting that PS-NPs may impair the ability of macrophages to produce NO. Collectively, our results suggest that NO plays a minor role in the increased bactericidal activity of macrophages exposed to PN-NPs.

### 3.4. Itaconate Mediates PS-NPs-Induced Bactericidal Activity

**Figure 6:**
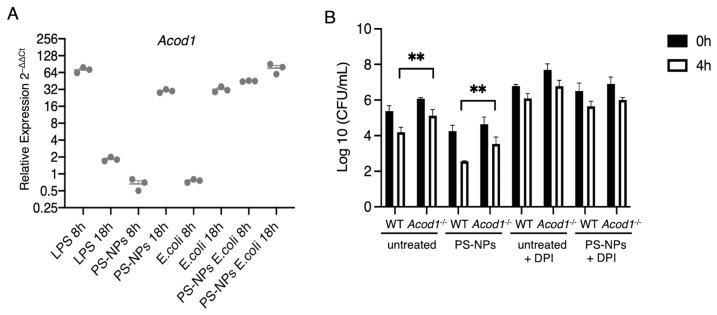
*Acod1* expression and bactericidal activity of PS-NPs-exposed BMM. (**A**). iBMMs were incubated with either LPS (100 ng/mL), PS-NPs (1 μg/mL), or *E. coli* (20:1) for 8 h and 18 h. Additionally, iBMMs exposed to PS-NPs for 18 h were incubated with *E. coli* (20:1) for 8 h and 18 h. *Acod1* levels were determined by quantitative RT-PCR. Data are represented as mean ±SEM. (**B**). BMMs from WT and *Acod1*^−/−^ mice (untreated or exposed to 1 μg/mL PS-NPs for 18 h) were incubated with *E. coli* (20:1). Where indicated, 10 μM DPI was added 1 h prior to the addition of *E. coli*. Bactericidal activity was determined by counting CFU at 0 h and 4 h post-treatment with gentamycin. Results are representative of three independent experiments performed in triplicate. Data are presented as mean ± SEM. ** *p* < 0.01.

Pro-inflammatory stimuli such as LPS induce the expression of cis-aconitate decarboxylase (ACOD1), which catalyzes the conversion of aconitate to itaconate [36]. In addition to its anti-inflammatory properties, itaconate contributes to the antimicrobial activity of macrophages [37,38,39]. We therefore sought to investigate the possible impact of PS-NPs exposure on the expression of *Acod1*. Figure 6A illustrates the relative expression levels of the *Acod1* in iBMM exposed to either LPS, PS-NPs, *E. coli*, or the combination of PS-NPs-*E. coli* at 8 h and 18 h post-exposure. Whereas LPS induced a strong but transient upregulation of *Acod1*, exposure to PS-NPs or *E. coli* led to increased and sustained *Acod1* expression over 18 h. Strikingly, exposure to PS-NPs and *E. coli* induced a strong expression of *Acod1* at 8 h post-exposure, which further increased at 18 h. This suggests an additive effect of the PS-NPs and *E. coli* in inducing robust *Acod1* expression. Having shown that the combination of PS-NPs and *E. coli* induced high levels of *Acod1* expression, we next assessed the potential impact of itaconate on the bactericidal activity of BMMs. To this end, wild-type (WT) and *Acod1*^−/−^ BMMs were either exposed to *E. coli* alone or to the combination of PS-NPs and *E. coli*. As found with iBMMs, WT BMMs exposed to PS-NPs exhibited significantly enhanced bactericidal activity compared to untreated BMMs (Figure 6B). In the absence of *Acod1,* we observed a significant reduction in the bactericidal activity of both untreated and PS-NPs-exposed BMMs (Figure 6B), consistent with a role for itaconate in mediating bactericidal activity of macrophages [37,38]. Inhibition of the NADPH oxidase with DPI markedly attenuated bacterial clearance in both WT and *Acod1*^−/−^ BMM exposed to PS-NPs or not (Figure 6B), suggesting that both NADPH oxidase-mediated ROS production and itaconate contribute to the enhanced bactericidal activity induced by PS-NPs.

## 4. Discussion

**Figure 7:**
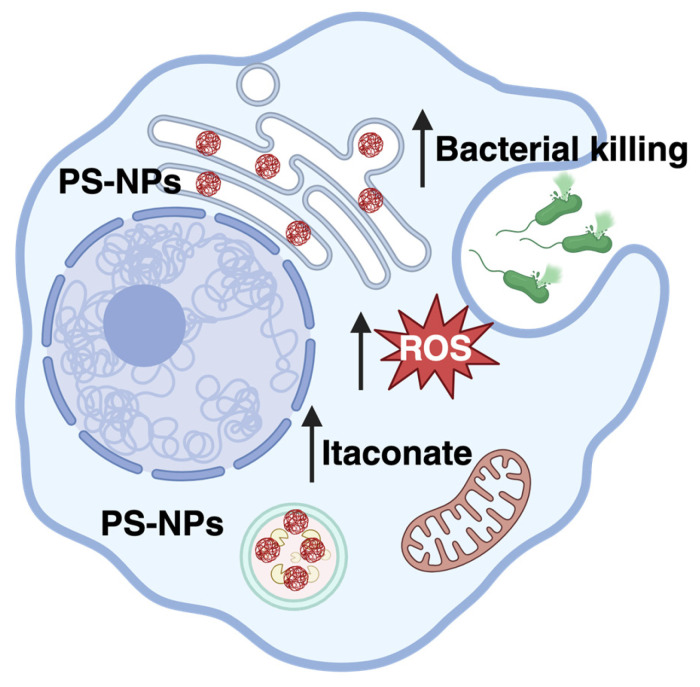
Exposure of macrophages to PS-NPs enhances the production of ROS and of itaconate, which contribute to enhanced bactericidal activity. Created in BioRender. Descoteaux, A. [2026] https://BioRender.com/z7lxrtz.

This study aimed at investigating the localization of PS-NPs in macrophages, as well as at studying their potential impact on the bactericidal activity of these cells. We obtained evidence that, in iBMMs, PS-NPs accumulate in the endo-lysosomal compartment, as well as in the ER. Moreover, we showed that pre-exposure to PS-NPs increases the bactericidal activity of macrophages. Further, we observed that ROS generated by the NADPH oxidase and the Krebs-cycle-derived metabolite itaconate both contribute to the increased bactericidal activity of PS-NPs-exposed macrophages. These data provide insight on the potential impact of NPs on the function of innate immune cells. These findings are summarized in Figure 7.

Owing to their small sizes, nanoparticles are readily internalized through endocytosis by macrophages [14,40]. At concentrations of 1 and 2 μg/mL used in our study, PS-NPs were not cytotoxic to iBMMs, although, at concentrations of 5 μg/mL and above, there is a slight but significant toxicity. This is in contrast to a recent study using Caco-2 cells which showed no decrease in cell viability when exposed to 80 nm PS-NPs at concentrations of up to 100 μg/mL [41]. Similarly, viability of A549 lung cancer cells exposed to 80 nm and 100 nm PS-NPs decreased at concentrations above 100 μg/mL [42,43]. The superior sensitivity of iBMMs towards PS-NPs compared to Caco-2 and A549 cells might reflect intrinsic cell type differences and remains to be elucidated. Interestingly, a comparison of silver NPs of different sizes revealed that smaller NPs were more toxic than larger ones for macrophages and fibroblasts [44]. Intriguingly, we observed a significant increase in the number of cells exposed to 1 μg/mL PS-NPs for 24 h, suggesting that this concentration PS-NPs stimulated iBMM proliferation. Studies with metallic NPs indicated that these particles may stimulate or suppress stem cell proliferation through various mechanisms [45]. How 1 μg/mL PS-NPs stimulates iBMM proliferation remains to be investigated. As previously reported in several cell types exposed to NPs of various compositions [14,18], we found that PS-NPs accumulated mainly in the endo-lysosomal compartment as well as in the ER. The consequences of PS-NPs accumulating within those intracellular compartments remains to be fully elucidated. In the case of endosomes and lysosomes, transiting of particles such as microbes through these compartments normally leads to their digestion and elimination. Lysosomal accumulation of undigestible material may impair the function of those degradative compartments, as previously shown in the murine microglial cell line BV2 exposed to PS-NPs [46,47]. In the case of the ER, it is unclear whether PS-NPs reach this central organelle through vesicular trafficking or through direct contact. Given the central role played by the ER in protein and lipid synthesis and transport, as well as carbohydrate metabolism [48], it is reasonable to assume that accumulation of PS-NPs in this organelle may affect its function. Further investigations will be necessary to elucidate how PS-NPs traffic to the ER and to understand their impact on its function.

An important consequence of NPs exposure in different cell types, including macrophages, is the generation of ROS [14,21,49]. For instance, polyethylene microplastics increase ROS generation in the macrophage-like cell lines U937 and THP-1 [50]. Similarly, polyethylene terephtalate NPs were reported to induce ROS production in the murine macrophage cell line RAW264.7 [51]. BMMs exposed to PS-NPs are no exception, as we detected the production of cellular and mitochondrial ROS. However, the exact mechanism through which PS-NPs induce ROS production in macrophages is not well understood. Disruption of mitochondrial function and impairment of the electron transport chain and activation of inflammatory responses are part of the mechanisms that have been proposed to contribute to the generation of ROS [21,52,53]. Production of ROS is central to the antimicrobial activity of macrophages [54]. Our finding that exposure to PS-NPs increased the bactericidal activity of BMMs raised the possibility that it was mediated by ROS produced in response to PS-NPs. Using the NADPH oxidase inhibitor DPI, we obtained evidence that ROS produced by the NADPH oxidase play a significant role in the increased bactericidal activity of PS-NPs-exposed BMMs.

Several studies have investigated the role of itaconate in host–pathogen interactions [37,55]. The ACOD1/itaconate axis in macrophages is induced by various inflammatory stimuli such as bacteria and LPS, indicating a role for itaconate in infection. ACOD1 is a mitochondrial enzyme which uses *cis*-aconitate from the Krebs cycle to synthesize itaconate in a single enzymatic step. Itaconate is exported from the mitochondria to the cytosol and transported from the cytosol into phagosomes, where it gets in contact with ingested bacteria. Itaconate kills bacteria through a few mechanisms, including its activity as a bacterial isocitrate lyase inhibitor [37,55]. In this context, our observation that exposure to PS-NPs alone or in combination to *E. coli* stimulated high levels of *Acod1* expression suggested that itaconate contributes to the increased bactericidal activity of BMM exposed to PS-NPs. This possibility was validated using PS-NPs-exposed *Acod1*^−/−^ BMM, which were significantly less bactericidal than PS-NPs-exposed WT BMMs. Interestingly, itaconate was shown to promote the production of ROS by the macrophage cell line RAW 264.7 in response to LPS [56]. In normal macrophages, LPS promotes the pentose phosphate pathway by increasing expression of the glucose-6-phosphate dehydrogenase and of the 6-phosphogluconate dehydrogenase. This leads to an increased production of NADPH, an intermediate metabolite of the pentose phosphate pathway, and to an up-regulation of the NADPH oxidase activity [56]. In LPS-stimulated *Acod1*^−/−^ RAW 264.7 cells, there was no increase in the expression of the glucose-6-phosphate dehydrogenase and of the 6-phosphogluconate dehydrogenase and no up-regulation of NADPH oxidase activity to produce ROS [56]. Consistently, we found that the bactericidal activity of WT and *Acod1*^−/−^ BMMs was inhibited to a similar extent by DPI. Clearly, further studies will be required to better understand the contribution of itaconate in macrophages exposed to PS-NPs. Furthermore, given the role of itaconate in the modulation of immune responses and inflammation [36,37,55], it will be essential to assess the potential impact of itaconate in in vivo models of NPs exposure.

## 5. Conclusions

In this study, we found that exposure of macrophages to PS-NPs stimulated ROS production and induced *Acod1* expression, leading to an increased bactericidal activity. Future studies will be necessary to investigate the impact of other NPs of different compositions which are increasingly present in the environment, including polyethylene, polyvinyl chloride, polypropylene, and polyethylene phthalate, on the microbicidal activity of macrophages.

## Figures and Tables

**Table 1:** Properties of the fluorescent PS-NPs used in this study in water and DMEM supplemented with 10% FBS.

Sample	Size (nm)	Hydrodynamic Diameter (nm)	PDI	ζ Potential (mV)
PS-Fluoro-Max Blue in H_2_O	50.83 ± 0.99	47.95 ± 0.48	0.106 ± 0.040	−14.3 ± 0.46
PS-Fluoro-Max Blue in DMEM + FBS	33.10 ± 1.75	16.80 ± 0.29	0.380 ± 0.006	−6.05 ± 2.39
DMEM + FBS	23.06 ± 0.69	14.82 ± 0.26	0.269 ± 0.001	−8.00 ± 0.97

**Table 2:** DLS analysis of DMEM supplemented with 10% FBS containing PS-Fluoro-Max Blue or not.

Sample	Peak 1 (FBS) (%)	Size 1 (FBS) (nm)	Peak 2 (PS-NPs) (%)	Size 2 (PS-NPs) (nm)
PS-Fluoro-Max Blue in DMEM + FBS	60.7	13.92	39.3	57.87
DMEM + FBS	100	22.27	0	N.D.

N.D. not detected.

## Data Availability

Data will be made available upon request.

## Abbreviations

The following abbreviations are used in this manuscript:

NO	Nitric oxide
ROS	Reactive oxygen species
BMMs	Bone marrow-derived macrophages
NPs	Nanoplastics
PS	Polystyrene
LPS	Lipopolysaccharide
ER	Endoplasmic reticulum
